# Impact of the Interprofessional Collaborative Education Program (IPCEP) on the knowledge, attitudes, and practices of diabetic foot care among individuals with type 2 diabetes mellitus: a narrative review

**DOI:** 10.1186/s12913-025-13244-0

**Published:** 2025-08-25

**Authors:** Bhukya Nom Kumar Naik, Sushma Prabhath, Elsa Sanatombi Devi, Arun G. Maiya, S. S. Prasad, Suvarna Hebbar, Gagana Karkada, Sahana Shetty, Shubhada Karanth

**Affiliations:** 1 Department of Anatomy, Kasturba Medical College Manipal, Manipal Academy of Higher Education (MAHE), Manipal, Udupi, Karnataka 576104 India; 2 Department of Anatomy and Medical Education, Kasturba Medical College Manipal, MAHE-FAIMER International Institute for Leadership in Interprofessional Education (M-FIILIPE)- FAIMER Regional Institute (FRI), Manipal Academy of Higher Education (MAHE), Manipal, Udupi, Karnataka 576104 India; 3 Department of Medical-Surgical Nursing, Manipal College of Nursing, Manipal, Manipal Academy of Higher Education (MAHE), Manipal, M-FIILIPE (FRI), Centre for Continuing Education and Interprofessional Development (CCEID), Udupi, Karnataka 576104 India; 4 Department of Physiotherapy, Manipal College of Health Professions, Manipal, Manipal Academy of Higher Education, Manipal, Centre for Diabetic Foot Care and Research (CDFCR), Udupi, Karnataka 576104 India; 5 Department of Surgery, Kasturba Medical College Manipal, Manipal Academy of Higher Education (MAHE), Manipal, Udupi, 576104 Karnataka India; 6 Department of Clinical Nutrition and Dietetics, Manipal College of Health Professions, Manipal, Manipal Academy of Higher Education, Manipal, Udupi, Karnatak 576104 India; 7 Institute for Molecular Medicine and Infectious Disease, and Department of Microbiology, Drexel University College of Medicine, Center for Molecular Virology and Gene Therapy, Philadelphia, PA United States of America; 8 Department of Endocrinology, Kasturba Medical College Manipal, Manipal Academy of Higher Education (MAHE), Manipal, Udupi, Karnataka 576104 India; 9 Department of Medicine, Kasturba Medical College Manipal, Manipal Academy of Higher Education (MAHE), Manipal, Udupi, Karnataka 576104 India

**Keywords:** Interprofessional collaborative education program (IPCEP), Knowledge, Attitude and practice, Type 2 diabetes mellitus, Diabetic foot Self-Management

## Abstract

**Background:**

Individuals with diabetes are at high risk of foot complications such as ulcers and amputations, leading to morbidity and economic burden. Effective foot care management reduces complications, highlighting the need for patient education and self-management practices. Interprofessional care (IPC) enhances patient care by integrating expertise from multiple disciplines. While education programs improve self-care and knowledge, their impact on self-efficacy remains unclear. Furthermore, the role of the Interprofessional Collaborative Education Program (IPCEP) in enhancing foot care practices also needs exploration.

**Objective:**

This narrative review evaluated the usefulness of the interprofessional approach (IPA) to patient-centered education in improving foot care knowledge, attitudes, and practices (KAPs) among individuals with type 2 diabetes mellitus (T2DM).

**Methods:**

A systematic search of PubMed, Scopus, and Cochrane was carried out to explore the importance of IPCEP in improving foot care practices among individuals with T2DM. A narrative review was conducted, as there was a paucity of literature related to studies meeting the criteria by September 23, 2024. Studies assessing patient outcomes (behaviour, attitudes, knowledge) were considered.

**Results:**

Extensive research is needed to highlight the importance of IPCEP in improving foot care knowledge and promoting proactive self-management. This is because the IPA positively influences patients’ attitudes toward health management post-education.

**Conclusion:**

This review highlights the need for IPCEP to improve foot care practices; thus, it can foster a holistic approach to diabetes and diabetic foot care, improve quality of life, and reduce complications.

## Introduction

Diabetic foot ulcers (DFUs) are a significant complication of T2DM, a long-term condition with substantial global health consequences. DFUs contribute to a high rate of nontraumatic lower limb amputations and largely affect the diabetic population. It is estimated that between 15% and 25% of diabetic people have DFUs [1–4]. Peripheral neuropathy, peripheral vascular disease, and poor glycemic management result in decreased blood flow (ischemia) and nerve damage (neuropathy) that results in ulceration of the feet [1, 4–7], which are frequently worsened by infections [2, 6–8]. People with type 2 diabetes can benefit greatly from diabetes self-management education (DSME), which gives them the knowledge and skills they need to control their disease properly.

DSME programs can significantly improve their effectiveness by integrating an IPA that involves various healthcare professionals (HCPs), including pharmacists, dietitians, and nurses. This collaborative method results in enhanced health outcomes by ensuring comprehensive support and care [9–11]. Interprofessional education (IPE) and teamwork-focused learning are vital in managing T2DM, as they strengthen healthcare providers’ ability to collaborate effectively, leading to improved patient outcomes. Numerous studies highlight the advantages and challenges of implementing IPE in diabetes management [12–14]. Addressing collaboration barriers and adopting formal IPE programs are essential steps for enhancing patient and clinical outcomes [15–19].

Focusing on enhancing patient outcomes through collaboration, multidisciplinary care, and interprofessional collaboration or approaches are vital elements of modern healthcare. Nevertheless, certain individuals may struggle to differentiate between ‘interprofessional care’ and ‘multidisciplinary care’. Both approaches involve multiple health professionals. The main distinction lies in the degree of collaboration and integration.

Table 1 describes the key differences between Interprofessional Care (IPC) and Multidisciplinary Care (MDC), highlighting how IPC emphasizes integrated teamwork and shared decision-making, while MDC typically involves professionals working independently within their own roles. To further clarify these concepts, commonly accepted definitions and examples are summarized in Table 2 below.

**Table 1:** Table comparing the differences between multidisciplinary care (MDC) and interprofessional care (IPC)

**Parameter**	**Multidisciplinary Care (MDC)**	**Interprofessional Care (IPC)**
Definition	Independent work by different professionals [20, 21]	Collaborative learning and practice among professions [22, 23]
Team Structure	Parallel, independent work [20]	Integrated, collaborative work [22–24]
Benefits	Improved patient outcomes, cost-effective [21]	Enhanced team dynamics better patient outcomes [20, 23, 24]
Challenges	Fragmented care due to limited interaction [20]	Requires strong organizational support [24, 25]

**Table 2:** Commonly Accepted Definitions and Examples of Collaborative Healthcare Concepts

**Term**	**Definition**	**Example**	**Key Benefit**
Multidisciplinary Care (MDC)	Multiple HCPs from different disciplines working independently on different aspects of a patient's care are involved. Each professional contributes expertise but typically works in parallel rather than collaboratively [26, 27].	A patient with cardiorenal syndrome might see a cardiologist and a nephrologist separately, each providing specialized care.	Utilizes specialized expertise for individual aspects of care.
Interprofessional Care (IPC)	Health professionals from different disciplines working together as a cohesive team to provide comprehensive care. This approach ensures that each team member's unique perspective contributes to a holistic understanding of the patient's needs [28, 29].	An integrated diabetes care team is vital for managing uncontrolled T2DM. This team includes an endocrinologist, dietitian, podiatrist, pharmacist, and psychologist. The endocrinologist adjusts medications for glycemic control, whereas the dietician provides tailored nutritional advice. The podiatrist performs foot exams to prevent complications, and the pharmacist ensures medication adherence. The psychologist addresses emotional distress and enhances coping skills. This teamwork improves outcomes and quality of life	Improving patient outcomes through integrated care plans. Enhanced patient engagement and satisfaction [28, 29].
Interprofessional Approach (IPA)	A method of healthcare delivery where multiple health workers from different professional backgrounds work together with patients, families, and communities to deliver the highest quality of care [29–31].	In a diabetic foot care, an interprofessional team might include an endocrinologist, podiatrist, neurologist, surgery specialist (vascular surgeon), diabetic nurse, physical therapists, dietician, and social workers working together to create or deliver high-quality care.	Holistic care for chronic and complex conditions.
Interprofessional (IP) Collaboration	Collaborative care provided by multiple HCPs who work together to deliver the highest quality of care to patients [32, 33].	A pediatric care team consisting of doctors, nurses, psychologists, and social workers working together to manage a child's health	Strengthens communication and shared responsibility in care.
Interprofessional Education (IPE)	Interprofessional education (IPE) is a collaborative teaching and learning approach in which students from two or more health professions learn together to improve teamwork and the quality of care [34–36].	Simulations, escape rooms, and collaborative problem-solving exercises could help improve IPE implementation through the development of teamwork, communication, and conflict-resolution skills [36, 37].	Prepares students for effective team-based, patient-centered practice. It also enhances teamwork and communication among HCPs.

### Rationale for the review

This review emphasizes the need for better management of T2DM and its complications, particularly diabetic foot issues, which burden patients and healthcare systems. Gaps in healthcare practices often lead to poor outcomes, including ulceration and amputation. Given these challenges, there is a growing recognition of the importance of educational interventions that not only inform but also empower patients in their self-care practices. It highlights the role of Interprofessional Collaborative Education Programs (IPCEP) in improving knowledge, attitudes, and practices related to foot self-care. Such programs emphasize the value of team-based, patient-centered approaches that integrate the expertise of various healthcare professionals. This review aims to evaluate the usefulness of the interprofessional approach (IPA) to patient-centered education in enhancing foot care knowledge, attitudes, and practices (KAPs) among individuals with T2DM. By fostering team-based care, IPCEPs attempts to bridge the gap between education and self-management. Therefore, examining the effectiveness of IPCEPs is essential in understanding their potential to strengthen foot care practices and reduce the incidence of diabetic foot complications. The review examines key issues like diabetes management complexity, KAP gaps, and healthcare challenges, highlighting how IPCEPs can improve clinical outcomes in diabetic foot care.

## Methodology

**Fig. 1:**
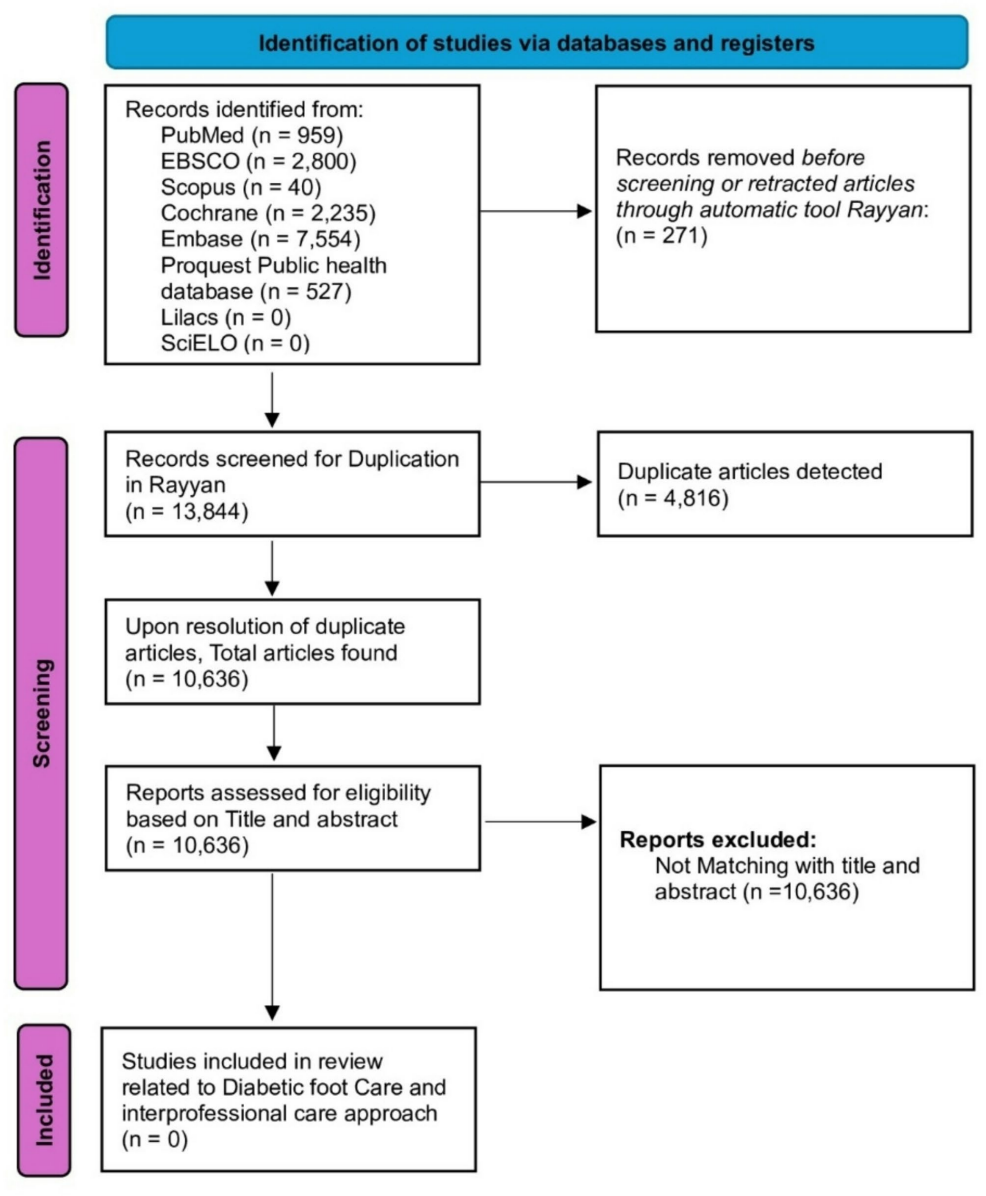
PRISMA flow diagram of the literature-selection process

A systematic search of articles archived in the PubMed, EBSCO, Scopus, Cochrane, Embase, ProQuest Public Health, Lilacs, and SciELO databases via keywords such as Interprofessional Education, Health Knowledge, Attitude, Practice, Diabetic Foot, Self-Care, Self-Management, and Diabetes Mellitus, Type 2, was carried out from inception until September 23, 2024 (Table 3). The original studies published in the English language literature that provide insights into interprofessional education, healthcare knowledge, attitudes, practice, diabetic foot, self-management/self-care, and diabetes mellitus type 2, performed in either Conference Materials, Editor notes, Special collections, Academic Journals, Magazines, Books, e-books, clinical trials, clinical Answers, or Journal Publications, were included. A PRISMA flow diagram of the literature selection process is detailed in Fig. 1.

**Table 3:** Search strings with results from various databases (PubMed, EBSCO, Scopus, Cochrane, Embase, ProQuest Public Health Database, Lilacs, and SciELO)

**Database**	**Search string**	**Period of search**	**Number of results without any additional filters**
PubMed	Search: ((((("Interprofessional Education"[Mesh]) AND ("Health Knowledge, Attitudes, Practice"[Mesh])) AND ("Diabetic Foot"[Mesh])) AND ("Self Care"[Mesh])) OR ("Self-Management"[Mesh])) AND ("Diabetes Mellitus, Type 2"[Mesh]) (("Interprofessional Education"[MeSH Terms] AND "health knowledge, attitudes, practice"[MeSH Terms] AND "Diabetic Foot"[MeSH Terms] AND "Self Care"[MeSH Terms]) OR "Self-Management"[MeSH Terms]) AND "diabetes mellitus, type 2"[MeSH Terms]	Inception to 23/10/2024	959
EBSCO	TX ( interprofessional education or ipe or interdisciplinary education or interprofessional learning ) AND TX ( health knowledge, attitudes, practice or knowledge, attitudes, practice ) AND TX diabetic foot AND TX ( self-management or self-management or self-care or self care ) AND TX ( type 2 diabetes or type 2 diabetes mellitus or t2dm )	Inception to 23/10/2024	2,773
Scopus	Interprofessional Education AND Health Knowledge, Attitudes, Practice AND Diabetic Foot AND Self-Care OR Self-Management AND Diabetes Mellitus, Type 2	Inception to 23/10/2024	40
Cochrane	ID Search #1 Interprofessional Education #2 Health Knowledge, Attitudes, Practice #3 Diabetic Foot #4 Self-Care #5 Self-Management #6 Diabetes Mellitus, Type 2 #7 #1 AND #2 AND #3 AND #4 OR #5 AND #6	Inception to 23/10/2024	2,235
Embase	((((('interprofessional education'/exp) AND ('attitude to health'/exp)) AND ('diabetic foot'/exp)) AND ('self care'/exp)) OR ('self care'/exp)) AND ('non insulin dependent diabetes mellitus'/exp)	Inception to 23/10/2024	7,554
ProQuest Public health Database	(interprofessional education OR ipe OR interdisciplinary education OR interprofessional learning) AND (health knowledge, attitudes, practice OR knowledge, attitudes, practice) AND (diabetic foot) AND (self-management OR self management OR self-care OR self care) AND (type 2 diabetes OR type 2 diabetes mellitus OR t2dm)	Inception to 23/10/2024	845 (Only full text and Peer reviewed)
Lilacs	Interprofessional Education AND Health Knowledge, Attitudes, Practice AND Diabetic Foot AND Self Care OR Self-Management AND Diabetes Mellitus, Type 2	Inception to 23/10/2024	0
SciELO	Interprofessional Education AND Health Knowledge, Attitudes, Practice AND Diabetic Foot AND Self Care OR Self-Management AND Diabetes Mellitus, Type 2	Inception to 23/10/2024	0
Total			14,406

### Inclusion criteria

- Studies (articles or abstracts) explicitly focusing on interprofessional education (IPE) or collaborative approaches in the prevention, management, or treatment of diabetic foot complications.
- Studies that examine multidisciplinary or interprofessional team-based care models specifically involving diabetic foot care, highlighting teamwork, communication, or joint decision-making.
- Research conducted in any healthcare setting (hospital, community, academic) where diabetic foot care is approached through collaborative or team-based strategies.

### Exclusion criteria

- Studies that focus on general diabetes management without a clear and specific emphasis on diabetic foot care.
- Studies that describe collaborative care but do not involve multiple healthcare professionals or lack interaction among team members (e.g., parallel care rather than interprofessional collaboration).
- Articles addressing only the role or perspective of a single profession in diabetic foot or diabetes care, with no reference to collaboration or teamwork.
- Reviews, commentaries, or editorials that do not include original data or analysis related to interprofessional approaches in diabetic foot care.

Initially, we intended to conduct a systematic literature review, anticipating studies that directly assessed the influence of IPCEPs on the KAP of foot care among individuals with T2DM. However, due to the scarcity of such targeted studies identified through our systematic search, we adopted a narrative review approach. The Results section presents this gap clearly and highlights the need to bridge it. Consequently, the Discussion section is structured around insights drawn from IPE and IPA articles that, while not directly focused on diabetic foot care, offer relevant perspectives to inform future directions and bridge existing knowledge–practice gaps.

#### Data extraction and synthesis

As our review revealed that no studies directly focused on IPCEPs in relation to diabetic foot care practices, a detailed data extraction and synthesis section was not included. Instead, we have presented descriptive paragraphs in the discussion section of the available evidence with supporting side headings and a table (Table 4) that describes the diabetic foot care knowledge and practice levels among various populations worldwide, especially in developing countries.

#### Ethical considerations

This article is a narrative review based solely on analysis of previously published literature and does not involve any primary data collection from human participants. It is not the direct output of a research project requiring ethical approval. Therefore, no ethical approval code is applicable to this study.

## Results

Our comprehensive literature search identified a total of 14,406 articles across various databases (Table 3). After removing duplicates, 10,636 articles remained for title and abstract screening. This rigorous selection process (Fig. 1), guided by predefined inclusion and exclusion criteria, ensured that only relevant and high-quality studies were considered.

The review highlighted a significant gap in the literature regarding the impact of IPCEP in improving KAP related to diabetic foot self-management among patients with T2DM. Notably, very few studies have specifically addressed this intersection, indicating an urgent need for further research.

Considering the scarcity of data specifically evaluating the impact of IPCEP on diabetic foot care, we undertook a narrative review to synthesize the available evidence and explore insights from related fields. Initially, we intended to conduct a systematic literature review, anticipating studies that directly assessed the influence of IPCEP on the KAP of foot care among individuals with diabetes. However, our systematic search revealed a lack of such targeted research, reflecting that interprofessional collaboration in diabetic foot care remains an emerging area.

Given this limitation, we adopted a narrative review approach to explore the potential role of IPCEP in enhancing self-management practices for diabetic foot care among individuals with T2DM. The current review not only highlights existing findings but also identifies critical research gaps and proposes directions for future investigations. By addressing this overlooked aspect of diabetes management, we aim to contribute to enhancing patient outcomes and developing more effective healthcare management strategies.

## Discussion

This narrative review highlights the need and impact of IPCEP on diabetic foot care KAPs among individuals with T2DM. Findings suggest that interprofessional collaborative approaches enhances patient knowledge, fosters positive attitudes, and improves self-care practices, ultimately reducing complications such as diabetic foot ulcers (DFUs) and amputations.

Diabetic foot complications are a major concern because of their high prevalence and severe impact on patients’ lives. DFUs affect up to 34% of the global diabetic population, contributing to significant morbidity, reduced quality of life, and high healthcare costs [38–40]. Preventative measures, early detection, and a multidisciplinary approach are key to managing these complications effectively [41–44]. As the treatment of DFUs is costly, especially in resource-poor settings, preventive care is essential to lowering healthcare expenses and improving patient outcomes [38, 45, 46].

Effective foot care, including regular screening, glycemic control, smoking cessation, and meticulous foot care, can prevent ulcers from progressing to severe stages and reduce the risk of amputation [5, 47, 48]. Research has shown that organized educational programs significantly improve the understanding of foot care standards and enhance the knowledge of effective foot care practices [49–52]. These educational interventions have been successful in fostering better foot care behaviors among patients. Those who received such education tended to adhere more closely to foot care routines, including regular foot inspections and proper personal hygiene [49–53].

A considerable gap exists between knowledge and actual foot care practices among individuals with T2DM. Many studies highlight that while a significant portion of diabetic individuals possess adequate knowledge about foot care, this knowledge often does not translate into good practices. Table 4 describes diabetic foot care knowledge and practice levels among various populations worldwide, especially in developing countries.

**Table 4:** Comparison of diabetic foot care knowledge and practices followed across developing countries

**Country**	**Location**	**Good Knowledge (%)**	**Good Practice (%)**
Kuwait [54]	-	79.3%	30.8%
Ethiopia [55]	Eastern Ethiopia	52.5%	20.2%
Nigeria [56]	-	30.1%	10.2%
China [57]	**-**	82.7%	22.4%
Saudi Arabia [58]	Madinah	35%	27%
Saudi Arabia [59]	Makkah	27.6% (Poor Knowledge)	63.3% (Poor Practice)
India [60]	Belagavi, South India	64.2%	16.9%
India [61]	Rural Haryana	63.5%	24% had Poor Knowledge
Jordan [62]	-	>50%	6%
Pakistan [63]	-	29.3%	14%

While knowledge levels vary globally, they are generally acceptable, with good knowledge rates ranging from 63.5–88% [61, 64]. Despite varying levels of knowledge, the practice of foot care remains inadequate across these developing countries (Table 4). However, a substantial gap exists between knowledge and practice, with fewer patients implementing proper foot care routines [65, 66]. Common foot care practices include washing, drying, moisturizing, and nail trimming, but many patients neglect crucial steps, such as checking for ulcers and choosing appropriate footwear [64]. These findings highlight the need for comprehensive educational programs and targeted strategies to improve foot care practices among diabetic patients globally.

To prevent diabetic foot ulcers (DFUs), raising awareness among diabetic individuals regarding foot care practices and early warning signs is crucial. Many health complications can be prevented because a substantial number of people are uninformed about the risks or proper methods of foot care [41, 67]. Providers and patients in healthcare need to be informed about foot care, which encompasses risk factors, appropriate hygiene, early indicators of DFU [5, 68, 69], and teamwork in care. IPE aims to overcome the barriers in healthcare by promoting collaboration among healthcare providers, ensuring that accurate information and care are delivered to patients. In addition to enhancing patient outcomes, this cooperative or collaborative method lowers the chance of complications such as DFU [41, 67].

Tailored education is crucial for efficient self-care and lowering DFU incidents [70–72]. It is essential to have a multidisciplinary team that includes podiatrists, diabetologists, and vascular surgeons to manage DFUs and lower amputation rates. This approach facilitates comprehensive care and considers the multifactorial nature of DFUs [73, 74]. IPE enhances collaboration among HCPs and positive perspectives on foot care by emphasizing the importance of teamwork and comprehensive treatment [75, 76]. This team-based method results in enhanced quality of care, prompt interventions, and better patient support and education [77], which are necessary to decrease the chances of serious complications, such as amputations, thus leading to better health outcomes and reduced healthcare costs [4, 21, 24, 30, 33]. Given the differences in study designs and findings, it remains uncertain whether education has a direct influence on clinical outcomes such as ulceration and amputation rates, even though studies indicate that education increases knowledge and behaviors among participants [50, 53].

To further enhance the effectiveness of IPCEPs in addressing the challenges of diabetic foot care, it is imperative to explore key components that influence program success. The subsequent subsections will delve into these essential elements that can significantly impact the effectiveness and overall outcomes of IPCEPs in managing diabetic foot care.

### Complexity of diabetes management

Diabetes cannot be managed just with the prescription of drugs; there is also a need to integrate medical, nutritional, psychological, and social support to improve patient adherence and reduce the associated complications. Such a condition allows for only an interdisciplinary approach in which HCPs combine efforts to address multifaceted aspects. In delivering comprehensive care, teamwork is crucial to ensure optimal patient outcomes.

### Challenges in contemporary healthcare practices

There are many challenges faced by contemporary healthcare practices, which affect the quality and consistency of care. One such challenge is that HCPs usually work in isolation, which results in poor communication and inconsistent care [69, 78, 79]. This can be confusing for patients because of the conflicting advice given and lower adherence to treatment plans [79, 80]. Shortages in the workforce and the complexity of chronic care exacerbate these communication gaps [69, 81, 82]. These challenges can be addressed through a systematic approach, such as IPE Programs, to improve collaboration among health care providers [78, 83].

### Bridging gaps in patient outcomes

A persistent gap in patient understanding, especially related to nutrition, lifestyle changes, and foot care, continues to be a major barrier in effective diabetes management. This knowledge gap often contributes to poor adherence to recommended health practices, which raises the risk of preventable complications [84–87]. Negative attitudes and psychological challenges, such as emotional distress, misunderstandings about treatment, and the burden of managing a chronic illness, further interfere with care [84–90]. Furthermore, poor self-management behaviors such as irregular self-care routines and difficulties in adopting healthier lifestyles contribute to preventable complications and suboptimal outcomes [84, 91–93].

Addressing these multifaceted challenges necessitates an IP approach that combines patient education, psychological support, and regular follow-up. By integrating these elements, IP collaboration can empower patients to manage their condition more effectively, reduce the risk of complications, and ultimately improve long-term health outcomes.

### Interprofessional education (IPE) as a solution

Effective IPE has been linked to improved patient outcomes, such as enhanced self-management and a reduction in clinical errors. A key advantage of IPE is its ability to improve the competence of healthcare providers and elevate patient care by promoting well-coordinated interprofessional care within teams that draw on diverse healthcare disciplines [12, 18, 94, 95]. For instance, IPE has been associated with notable improvements in the management of diabetes, including better glycemic control, blood pressure, and triglyceride levels [68, 96, 97].

However, while IPE offers numerous benefits, challenges remain in designing effective activities and breaking down professional boundaries. These challenges include competing interests across different healthcare professions, logistical constraints, and the need for substantial resources and support from educational institutions [94, 95, 98]. Despite these obstacles, the positive impact of IPE on collaboration and care quality in diabetes management is well-documented. Studies have consistently shown that IPE programs lead to improved patient outcomes, such as enhanced self-management behaviors, reduced hospital admissions, and a better quality of life for patients [12, 13, 97].

Implementing IPE can further enhance collaboration among providers, ensuring more coordinated care [78, 83, 99]. By improving teamwork, communication, and fostering a shared understanding of roles, IPE strengthens the quality of diabetic care [18, 45, 77, 100–103]. Involving community health workers within care teams can also contribute to better patient education and support, emphasizing the importance of improving teamwork and communication to address these issues effectively [69, 78, 79, 83].

### Integration of IP collaboration for better outcomes

Research on the use of IP collaboration in teaching diabetic foot self-management underscores the necessity of a team-based approach to enhance both patient outcomes and healthcare delivery [99]. Within this model, HCPs emphasize patient-centered care, ensuring that interventions are tailored to individual needs. An IP team brings together diverse expertise, addressing all aspects of diabetic foot care from prevention to treatment while enhancing communication, decision-making, and overall management. This collaborative approach fosters knowledge sharing, which in turn strengthens diagnosis, treatment, and patient education. Simultaneously, effective teamwork promotes time efficiency and optimal use of available resources.

To maintain the benefits of collaboration, a supportive learning environment and ongoing training are essential for sustaining both patient and provider motivation. However, successful implementation depends on active stakeholder engagement and the presence of supportive policy frameworks. While the advantages of IP collaboration are well recognized, HCPs also report barriers, including logistical challenges and restrictive policies. Tackling these through structured reforms can further solidify collaborative care. Ultimately, the findings demonstrate that IP collaboration not only enhances patient education and self-management but also improves overall care delivery and supports policy advocacy for the long-term management of diabetic foot complications [99].

### IPCEP and knowledge, attitude, & practice (KAP) in diabetic care

The role of IPCEP in improving KAP among individuals with Type 2 Diabetes Mellitus (T2DM) is of great significance. A key component of IPCEP is patient activation interventions, which, as shown in randomized controlled trials, lead to marked improvements in glycemic control and self-management behaviors (SMBs) such as physical activity, healthy eating, foot care, and blood glucose monitoring [104]. Complementing this, the multidisciplinary team approach within IPCEP strengthens collaboration, utilizes diverse clinical expertise, and promotes patient-centered decision-making to enhance the quality of diabetes care [105].

These programs further support KAP improvement by offering tailored and follow-up sessions that reinforce SMBs, as evidenced by the success of patient activation strategies [104]. Tools like the Directive and Nondirective Support Scale for Patients with Type 2 Diabetes (DNSS-T2DM) highlight the value of individualized support that aligns with each patient’s unique needs, thereby enriching the overall care experience [106]. However, challenges remain. Despite positive knowledge and attitudes, many individuals still struggle with poor self-care behaviors, pointing to the pressing need for structured and accessible educational interventions [106–108].

Evaluating the effectiveness of IPCEP requires assessing KAP levels through cross-sectional studies, which help uncover knowledge gaps and direct efforts toward high-risk groups, particularly those with lower education levels [109, 110]. Additionally, complementary interventions such as DiabeText–a mobile health (mHealth) program have demonstrated success in supporting diabetes self-management and improving clinical outcomes, suggesting that digital tools can enhance the reach and impact of interprofessional strategies [111].

### Importance of IPCEP in patient education for diabetic foot care

IPCEP enhances patient care by fostering collaboration among HCPs. Unlike traditional patient education methods, it ensures a team-based approach. Specialists work together, and all facets of the patient’s health are taken care of, which results in better patient participation, better education, and better outcomes [99].

With respect to patient outcomes, the IP approach has been shown to improve diabetic foot ulcer treatment and lower complications, leading to infections and, inversely, reduced amputation rates [112]. The integration of multiple professional perspectives leads to a more holistic approach, allowing for better issue detection and faster intervention. Reductions in the frequency of foot-related problems and faster healing periods for foot ulcers are proof that the all-encompassing character of the IP approach has improved patient outcomes [99, 113]. Additionally, through organized instructional modules, the IP approach to patient education, or IPE, offers ongoing encouragement, assistance, and adherence to foot care procedures over the long term, and these modules support patient involvement. Unlike other approaches, IPCEP creates a setting where patients are continuously reminded of critical self-management techniques [99].

Compared with other educational interventions, nurse-led programs, while beneficial for improving foot care practices and lowering neuropathy, frequently lack the interdisciplinary depth provided by IPCEP. Even though nurses are essential in the treatment of diabetes, the comprehensiveness of care may be limited if other medical experts do not contribute [47, 114]. On the other hand, written and interactive teaching materials can enhance patient outcomes. Written materials, for example, are effective in improving foot health ratings, whereas interactive sessions are especially effective in increasing confidence in preventive care practices. However, despite their benefits, these methods often do not provide the holistic and continuous support that IPE does, falling short in terms of sustainability [35, 79].

Another strategy that has been successful in increasing awareness and promoting better foot care habits is the use of audio-visual teaching modules [115]. Like written and interactive sessions, these approaches might not, however, provide the continuous and individualized reinforcement that patients need. Although they are helpful in short-term situations, patients can find it difficult to sustain these better habits over time without ongoing assistance from a multidisciplinary team [115].

The usefulness of IPCEP is evident in a study focused on the development and validation of the IPC educational module, which demonstrated notable success in advancing knowledge and self-management practices among individuals with T2DM, as observed in a pilot population [112]. Participants recognized its value in enhancing their understanding and practical implementation of diabetic foot care. The educational content and delivery method, particularly through WhatsApp, proved to be both accessible and engaging, catering to the convenience of learners. Tailoring the module to individual needs further strengthened learning outcomes. Moreover, consistent follow-ups and reminders played a key role in reinforcing behavioral changes, contributing to improved foot care routines. Over time, such interventions can significantly reduce hospital admissions, lower the risk of amputations and mortality, and ultimately improve the quality of life for individuals and society [112].

Building on these findings, IPCEP offers continuous support and integrates insights from various disciplines, making it a robust tool for long-term diabetic foot care management. The approach leverages the collective expertise of HCPs, which turns patient education into a personalized and essential experience [99, 113]. Long-lasting improvements in self-care behaviors are driven by the ongoing reinforcement of knowledge and habits core elements of the IPCEP framework [116]. Unlike single-discipline methods, interprofessional collaboration within IPCEP esnsures that diverse patient needs are comprehensively addressed [99]. Consequently, IPCEP holds a distinct advantage over traditional educational methods in managing diabetic foot complications [99, 113, 117].

Furthermore, IPCEP nurtures a collaborative environment that supports improved patient outcomes and more effective healthcare delivery [18, 45, 77, 100–103]. Programs like the Diabetes, Multidisciplinary, Experiential (DIAMANTE) initiative illustrate how interprofessional training can boost both the knowledge and confidence of healthcare providers, such as pharmacists, in diabetes management [100].

### Limitations

This review has a few limitations. First, there are very few studies that directly focus on IPCEP in diabetic foot care, which limits the strength of our conclusions. While the topic is of growing importance, IP collaboration in this context remains an emerging concept, and existing studies are limited in scope. The included studies also varied in design, participant characteristics, and reported outcomes, which made direct comparisons difficult. In addition, only English-language studies were included, which may have led to the exclusion of relevant research published in other languages. We also excluded broader diabetes foot care studies that did not focus specifically on IP approach, which may have narrowed the scope of our findings. Furthermore, most of the included studies lacked long-term follow-up and did not report on critical clinical outcomes such as ulcer healing, amputation rates, or hospital admissions.

## Conclusion

This review highlights the urgent need for IPCEP as a transformative strategy in diabetic foot care, offering a comprehensive, patient-centered approach that enhances self-management, prevents complications, and improves the quality of life for individuals with T2DM. By fostering seamless collaboration among HCPs, IPCEP strengthens communication, clinical decision-making, and interdisciplinary teamwork, leading to fewer amputations, reduced disease burden, and better long-term outcomes. To achieve sustained impact, integrating IPCEP into clinical practice, patient education, and healthcare policies is imperative. This shift is not just an enhancement but a necessary evolution in diabetes care, ensuring that evidence-based, collaborative strategies become standard practice in preventing and managing diabetic foot complications.

### Future directions/recommendations

While this review focused specifically on diabetic foot ulcers (DFUs) within the context of IP collaboration and patient education, future research could benefit from broader comparisons with other high-risk conditions such as vascular ulcers and CKD-related skin ulcers. Interdisciplinary management protocols in these conditions may offer transferable insights for DFU care.

Long-term studies are needed to understand the sustained impact of IPCEPs on diabetic foot care outcomes. Involving patients in IPE programs can enhance learning and provide healthcare providers with insights into real-world challenges in foot care management. Evaluating the effects of IPE on both HCPs and patient outcomes remains essential. Additionally, community-oriented IPCEP initiatives involving local healthcare personnel and outreach services should be explored to extend the benefits of education and improve care practices across diverse populations.

## Data Availability

No datasets were generated or analysed during the current study.

## Abbreviations

IPCEP: Interprofessional Collaborative Education Program
IP: Interprofessional
IPC: Interprofessional Care
IPA: Interprofessional Approach
IPE: Interprofessional Education
MDC: Multidisciplinary Care
HCPs: Healthcare Professionals
T2DM: Type 2 Diabetes Mellitus
KAP: Knowledge, Attitude, and Practice
DFUs: Diabetic Foot Ulcers
DSME: Diabetes Self-Management Education
SMB: Self-Management Behaviors
